# Dr. A. Wellington Adams

**Published:** 1881-01

**Authors:** 


Dr. A. Wellington Adams.—We were favored with a visit a few days ago, from Dr. A. Wellington Adams, editor of the *Rocky Mountain Medical Review.* We found our confrere an intelligent and accomplished gentleman, and an earnest worker in our calling. His journal is ably edited and its mechanical execution reflects great credit upon the enterprise of the new city and state, from which it issues. We cordially wish the editor and his journal much success.

